# Risk factors for addiction among patients receiving prescribed opioids: a systematic review protocol

**DOI:** 10.1186/s13643-017-0642-0

**Published:** 2017-12-28

**Authors:** Amber Cragg, Jeffrey P. Hau, Stephanie A. Woo, Christine Liu, Mary M. Doyle-Waters, Corinne M. Hohl

**Affiliations:** 1 Department of Emergency Medicine, University of British Columbia, 855 West 12th Avenue, Vancouver, BC V5Z 1M9 Canada; 2 Vancouver General Hospital, 855 West 12th Avenue, Vancouver, BC V5Z 1M9 Canada; 3 Department of Medicine, University of British Columbia, 2194 Health Sciences Mall, Vancouver, BC V6T 1Z3 Canada; 4 Centre for Clinical Epidemiology and Evaluation, Vancouver Coastal Health Research Institute, 828 West 10th Avenue, Vancouver, BC V5Z 1M9 Canada; 5 Emergency Department, Vancouver General Hospital, 855 West 12th Avenue, Vancouver, BC V5Z 1M9 Canada

**Keywords:** Medication safety, Opioid addiction, Risk factors, Opioid-naïve, Opioid prescribing, Opioid dependence, Opioid use disorder, Systematic review, Protocol

## Abstract

**Background:**

Opioid addiction prevention has become an urgent public health priority, with several countries declaring a state of emergency due to rising death tolls from opioid abuse. Reducing the risk of developing addiction among opioid-naïve patients exposed to prescribed opioids during the process of medical care may be an important primary prevention strategy. Our objective is to synthesize the available evidence about factors associated with the development of addiction among patients first exposed to prescribed opioids, with a focus on opioid-naïve patients.

**Methods:**

We will perform a systematic search of MEDLINE, Embase, Cochrane Central Register of Controlled Trials, and other databases in collaboration with a health information specialist using a comprehensive search strategy. We will also supplement our search with a scan of the grey literature to identify relevant ongoing and unpublished studies. We will include studies reporting on risk factors for opioid addiction in patients prescribed opioid analgesic therapy through a prescription from a licensed medical professional, with a focus on opioid-naïve patients. We will exclude studies focusing on patients who are first exposed to illicit opioids, those who use prescription opioids for cancer pain, and/or who are palliative. Two reviewers will independently review titles, abstracts, and full texts for inclusion and exclusion criteria. They will then extract data from included full texts using standardized piloted data extraction forms and assess study quality through risk of bias assessment. We will synthesize the effect sizes of risk factors derived from clinically homogenous studies with similar designs and the remaining ones qualitatively.

**Discussion:**

Understanding risk factors for opioid addiction among patients who require analgesia has the potential to inform clinical care and opioid prescribing guidelines aiming to reduce opioid addiction. We will also use this information as a starting point for developing interventions for primary prevention.

**Electronic supplementary material:**

The online version of this article (10.1186/s13643-017-0642-0) contains supplementary material, which is available to authorized users.

## Background

### Introduction

Prescription opioids have made pain associated with otherwise debilitating medical conditions treatable. Unfortunately, the medical use of opioids can lead to addiction, dependence, or non-medical use. Addiction is characterized by a strong desire to take the drug, difficulties in controlling its use, persisting in its use despite harmful consequences, a higher priority given to drug use than to other activities and obligations, increased tolerance, and sometimes a physical withdrawal state [1–3]. Addiction to prescription opioids is associated with transition to illicit opioid use like heroin [4], greater health services utilization [5, 6], and increased mortality [1, 7]. Illicit drug users are more likely to experience social isolation [8], incarceration and criminalization [9], homelessness [10], disability or unemployment [7], mental health illness, and acute and chronic infections [7, 11]. Opioid addiction prevention has become an urgent public health priority internationally, as the USA and Canada have experienced sharply rising death tolls from opioid overdoses [12, 13].

While policies to promote responsible opioid prescribing have been implemented across North America to prevent inappropriate use at the population-level [14, 15], opioids continue to be the mainstay of treatment for acutely painful medical and surgical conditions, such as fractures, or renal or biliary colic. Preventing opioid prescribing among opioid-naïve patients who are at high-risk of addiction to prevent their development of tolerance and transition into to long-term use and abuse may be helpful in primary prevention at the population-level. This could be done by prioritizing high-risk patients for regional anesthetic or other procedures to treat painful conditions for which wait times are usually lengthy, or by using alternative agents more aggressively in the acute setting (e.g., non-steroidal anti-inflammatories, ketamine). To identify high-risk patients to avoid or minimize the use of opioids in these patients, we must identify risk factors for subsequent addiction prior to patients’ initial exposure.

Two systematic reviews published before 2008 investigated the proportion and predictors of opioid misuse among chronic pain patients [16, 17]. One study found between 3.3–14.5% of long-term prescription-opioid users became addicted after an average exposure time of 22.1 months, indicating that the duration of opioid exposure was an important factor in the development of addiction [16]. In the other review, risk factors were inconsistently measured across studies and demonstrated mixed effects as predictors [17]. The latter study did not focus on opioid-naïve patients in whom risk factors may be different, and for whom primary prevention strategies need to be developed to prevent addiction [17].

### Objectives

Our main objective is to synthesize the available evidence about patient-, provider-, medication-, and system-level risk factors, as well as protective factors, for the development of opioid addiction among patients exposed to prescribed opioids, with a focus on opioid-naïve patients. We will focus on risk factors that are observable at the point-of-care and modifiable, in order to inform clinical care and the development of primary prevention strategies.

Specific objectives are to synthesize the available evidence on:

1. Patient-, provider-, medication-, and system-level risk factors for the development of opioid addiction, and their effect sizes overall, and in opioid-naïve patients, to understand factors that potentiate the subsequent development of addiction
2. The characteristics of the clinical indication, prescriber (e.g., practice location, specialty), and prescription (e.g., type of medication, dosage, quantity dispensed, length of exposure) of the initial opioid prescription after which patients develop addiction to understand the clinical context of the opioid exposure
3. The effect of varying addiction outcome definitions at follow-up on the identified risk factors and the strength of their association

## Methods

We will conduct a systematic review of the literature that will adhere to the PRISMA guidelines for the reporting of systematic reviews (Additional file 1: Appendix A) [18].

### Eligibility criteria

**Fig. 1:**
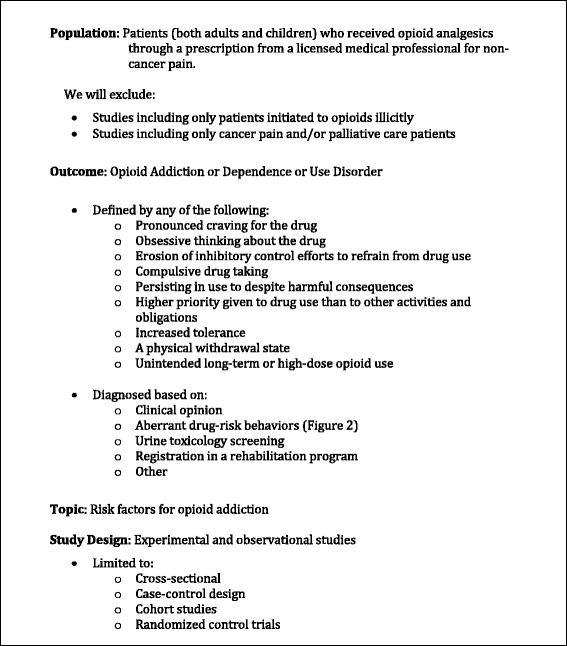
Study selection criteria

We adapted the Population, Intervention, Comparison and Outcome (PICO) framework for our systematic review, as we did not seek to synthesize information on the effectiveness of interventions. We will use Population, Outcome, Topic, and Study selection (POTS) to describe our study selection criteria (Fig. 1).

#### Population

We will include studies in which adults or children were first exposed, or report being first exposed to opioid therapy that was prescribed by a licensed medical professional. We will exclude studies in which all included patients were first exposed to illicit opioids. Studies with patients prescribed opioids for cancer pain or palliative care will also be excluded, as withholding analgesia from these patients is deemed unethical [19]. If studies do not report these baseline variables, we will include them, and perform sensitivity analyses on the effect of including these studies’ results on our review findings. If studies do not disaggregate the patient population based on these variables, we will attempt to contact study authors for patient-level data. If we are unable to access patient-level data, we will exclude studies in which more than 50% of patients meet our exclusion criteria, as these patients’ risk factors for developing addiction are likely different from patients first exposed to prescribed opioids. We will exclude studies reporting on patients on opioid receptor agonists used for non-pain indications, such as loperamide and dihydrocodeine.

#### Outcome

**Fig. 2:**
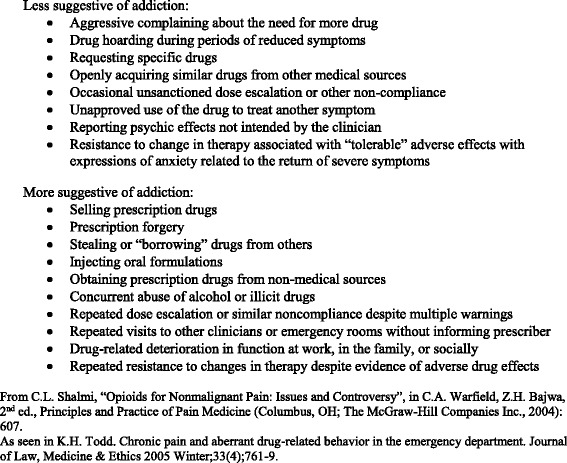
Included aberrant drug-related behaviors (ADB) [21, 23]

Opioid addiction has been defined as any of the following features: the pronounced craving for the drug, obsessive thinking about the drug, erosion of inhibitory control efforts to refrain from drug use, and compulsive drug taking [20]. As of 1964, the WHO has replaced the term “opioid addiction” with “opioid dependence” and defined it as “a cluster of behavioural, cognitive, and physiological phenomena that develop after repeated substance use and that typically include a strong desire to take the drug, difficulties in controlling its use, persisting in its use despite harmful consequences, a higher priority given to drug use than to other activities and obligations, increased tolerance, and sometimes a physical withdrawal state.”(ICD-10 definition) [3, 20, 21]. According to the DSM-5, opioid use disorder also includes taking the opioid in larger amounts or for longer than intended, not being able to cut down or quit, and spending a lot of time getting, using, or recovering from the use of the substance [20]. We will use the terms opioid addiction, opioid use disorder, and opioid dependence interchangeably and define them as evidence of any one of the features listed in any of the above definitions (Additional file 1: Appendix B). We will include studies that ascertain the outcome opioid use disorder, addiction, or dependence using any method presented in the literature, including but not limited to clinical opinion, evidence of aberrant drug-related behavior (ADB) (Fig. 2) [22], urine toxicology screening, and/or enrollment in a rehabilitation program [17].

### Topic

**Fig. 3:**
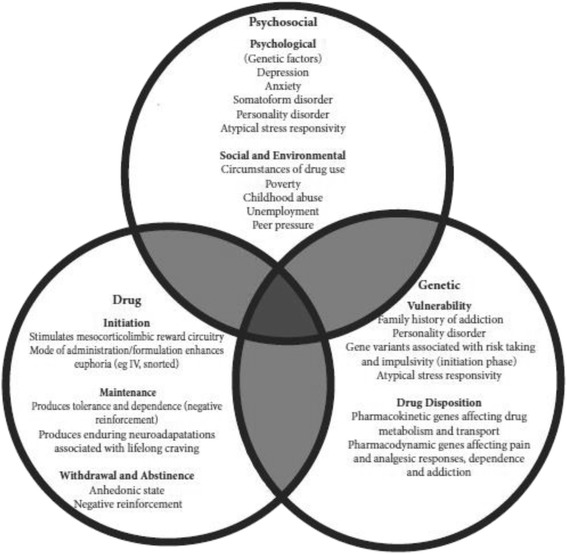
Potential risk factor categories [28]

We will include studies that report on at least one risk factor for opioid addiction. We define risk factor as an attribute, characteristic, or exposure that increases the likelihood of developing disease. Such factors will include patient-, provider-, medication-, and system-level risk factors, including well-known psychosocial and social factors, and will include protective factors (Fig. 3). Recent literature has indicated that previously unknown provider-level risk factors, such as the provider’s intensity of opioid prescribing, are related to subsequent addiction risk [23]. Medication-level factors such as the medication prescribed, and system-level risk factors such as the ability to dispense opioids in some healthcare settings, may also be associated with addiction risk. In our analysis, we seek to synthesize the available information on such factors, while emphasizing risk factors that are observable by clinicians at the point-of-care, as well as modifiable in order to produce information that will be directly relevant to clinical care activities and the development of preventative strategies.

#### Study design

We will include prospective and retrospective observational and experimental studies, including but not limited to randomized control trials and cross-sectional and case-control studies [24, 25].

### Search strategy

We will develop a systematic search strategy with a professional librarian (MDW). We will develop search terms and concepts by examining MeSH subject headings from six papers included in a prior systematic review [17]. These subject headings and an environmental scan of the literature will help us refine our search concepts. We developed a preliminary search in MEDLINE by combining the concepts *opioids* AND *pain* AND *risk factors* (Additional file 1: Appendix B). We will develop additional searches by expanding and combining different search concepts, including appropriate subject headings and keywords as needed. We will adapt searches for additional databases and will include studies published in English, French, and German after 1964, the year that the definition of “opioid dependence” was formalized by the WHO [3, 21].

### Information sources

We will search the following *electronic reference databases*: MEDLINE, Embase, Cochrane Central Register of Controlled Trials (CENTRAL), Database of Abstracts of Reviews of Effects (DARE) available through Ovid; CINAHL—Cumulative Index to Nursing and Allied Health Literature through EBSCO; and the Science Citation Index (Web of Science Core Collection) from Thomson Reuters. We will search social sciences databases including PsycINFO through EBSCO, Social Sciences Citation Index (Web of Science Core Collection) from Thomson Reuters, and the Sociology Collection through ProQuest. We will conduct *snowballing searches* for cited and citing studies of all papers meeting our inclusion criteria using the Web of Science Core Collection and ScienceDirect (Elsevier). We will also hand-search the bibliographies of relevant reviews and included studies for other potential titles for inclusion. We will search for *ongoing studies* by reviewing the following trial registries for unpublished trials, including the ISRCTN Registry, ClinicalTrials.gov, EU Clinical Trials Register and South African National Clinical Trials Register, Open Trials, and the Quebec Pain Registry. We will complete a *grey literature search* for additional unpublished studies using a combination of search terms and concepts derived from our electronic reference database search using the web search engine Google. We will review the top 100 hits for each search to identify relevant guidelines, reports, plain language publications, and websites of relevant professional associations. We will search for additional unpublished papers in the conference proceedings of the World Congress on Pain (IASP) and the International Conference and Exhibition on Pain Medicine and by looking through the table of contents for all published issues of Pain Medicine, Pain Research & Management, Anesthesia & Analgesia, and the Journal of Pain and Symptom Management since 1964 for relevant titles. We will search the websites of key medical associations, addiction, pain agencies (e.g., American Society of Addiction Medicine, National Institute of Drug Abuse, Chronic Pain Research Alliance), and government organizations (e.g., Centre for Disease Control, Health Canada) for additional unpublished literature and policy papers. Finally, we will contact study authors and experts in the field for additional unpublished studies.

### Data management

**Fig. 4:**
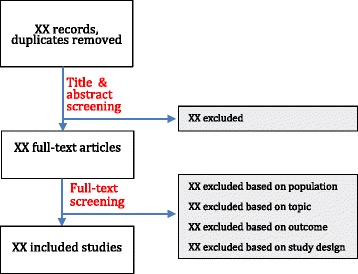
Study flow diagram

We will create a search report of all searches and their sources and capture the records of all eligible papers using RefWorks. We will use unique folders for each step of the search process within a common team RefWorks account (Fig. 4). We will de-duplicate search results using RefWorks and Excel. We will record the reason for exclusion for each record at the full text screening stage (Additional file 1: Appendix C).

### Selection process

Two reviewers will independently review titles and abstracts of all identified references for inclusion and exclusion criteria (Fig. 1). All potentially relevant titles identified by either or both reviewers will be moved forward for full-text review (Fig. 4). Two authors will independently review all potentially relevant full texts for inclusion and exclusion criteria (Fig. 4; Additional file 1: Appendix C). We will resolve disagreements relating to the inclusion or exclusion of full-text articles through discussion until we achieve consensus. If consensus cannot be reached, a third reviewer will adjudicate. Both reviewers will pilot test the inclusion and exclusion criteria data collection form on the first 100 search results to ensure we adequately describe and consistently apply the criteria (Additional file 1: Appendix C). One author will review the Google search result pages to collect relevant websites using combinations of the most pertinent search terms and will track the search terms, search engine used, and date of each search.

### Data collection process

Two reviewers will independently extract relevant data from each included study using a standard data extraction form (Additional file 1: Appendix D). We will resolve any disagreements through discussion until we achieve consensus. If consensus cannot be reached, a third reviewer will adjudicate. Both reviewers will pilot test the data collection form on the first five included studies (Additional file 1: Appendix D).

### Data items

We will collect information about the study design, methodology, participants, setting, prevalence of opioid addiction, potential and actual risk factors, timelines, and prevalence of opioid abuse. We will contact study authors for any missing information or clarifications required for data synthesis. We will attempt to contact authors by email a maximum of two times with the emails sent three weeks apart.

### Risk of bias in individual studies

Two reviewers will independently appraise each included study for potential sources of bias. We will assess the quality of observational studies using the NICE quality appraisal checklist for quantitative studies reporting correlation and associations (Additional file 1: Appendix E) and the NICE quality appraisal checklist for quantitative intervention studies (Additional file 1: Appendix F) [26]. Randomized control trials will be assessed using the Cochrane Risk of Bias Tool. We will assess each study for selection, performance, attrition, and reporting bias, and possible conflicts of interest using these tools. In the case of disagreement, two reviewers will discuss their rating until consensus is reached. If consensus cannot be reached, a third reviewer will adjudicate. We will perform subgroup analyses by the quality of the primary studies to assess how study quality affects our overall findings.

### Data synthesis

If we identify two or more clinically homogenous studies reporting effect sizes of the same outcome measure, we will synthesize the available information using random effects meta-analysis using RevMan5.3. We will report data on factors positively and negatively associated with opioid addiction using odds ratios along with their 95% confidence intervals. We will not pool data from studies of different designs, as their effect size estimates are expected to vary. We will assess heterogeneity using the *I* statistic [2]. If insufficient studies are found for meta-analysis, or studies are not homogenous, we will synthesize the data narratively.

### Sensitivity analysis

We will identify studies enrolling opioid-naïve patients and conduct a sensitivity analysis to identify their risk factors and effect size estimates in comparison to all included patients. For this purpose, we will define opioid-naïve patients, according to thresholds outlined by the Food and Drug Administration, as patients who have never taken daily opioid medications in excess of 60 mg oral morphine/day, 25 μg transdermal fentanyl/hour, 30 mg oral oxycodone/day, 8 mg oral hydromorphone/day, 25 mg oral oxymorphone/day, or an equianalgesic dose of another opioid for more than one week consecutively [27]. We will also perform sensitivity analyses on the route of first exposure to determine the effect of including studies in which some patients were first exposed to illicit opioids, were prescribed opioids for cancer pain, or were palliative. We will also conduct sensitivity analyses on the length of the initial opioid prescription, the outcome definition, and method and timing of addiction ascertainment.

### Confidence in cumulative evidence

We will present results of our meta-analysis using a GRADE summary of findings table, by method of outcome ascertainment. This table will present the summarized effect sizes alongside a score for the quality of the evidence used to generate that value. We will assign the quality of evidence scores (or GRADEs) based on the number and quality of the component studies and the consistency and generalizability within them. We will use funnel plots to assess for publication bias, if we have more than the necessary ten included studies.

## Discussion

### Dissemination of results and publication policy

We will disseminate the results of this project through traditional methods, including abstracts to national and international meetings, and peer-reviewed papers. We will produce patient-friendly summaries in lay language to disseminate relevant results to the public through our websites, in the bulletins of patient safety organizations and in the lay press. We will produce briefing notes for diverse knowledge user groups including healthcare managers and decision makers within healthcare institutions, patient safety organizations, and government.

### Limitations

The main limitation of our proposal is that we will only be able to perform meta-analysis if the risk factors measured in the primary studies are homogenous across studies. We will narratively synthesize all other data. Other limitations include the inclusion of publications in English, French, and German and quantitative publications only.

### Potential impact

Up-to-date information on risk factors for opioid addiction among patients receiving opioids has the potential to inform clinical care and opioid prescribing guidelines, and encourage the derivation and validation of screening tools to identify risk of addiction in patients being prescribed opioid analgesics.

## Additional file

Additional file 1: Appendix A. PRISMA-P 2015 Checklist [18]. Appendix B. MEDLINE Search from June 26, 2017. Appendix C. Inclusion/Exclusion Form. Appendix D. Data Collection Form. Appendix E. NICE Quality Appraisal Checklist for Quantitative Studies Reporting Correlations and Associations [26]. Appendix F. NICE Quality Appraisal Checklist for Quantitative Intervention Studies [26]. (DOCX 98.8 kb)

## Abbreviations

ADB: Aberrant drug-related behavior
WHO: World Health Organization

## References

[1] Paulozzi LJ (2012). Prescription drug overdoses: a review. J Saf Res.

[2] Wachholtz A, Gonzalez G, Boyer E (2011). Intersection of chronic pain treatment and opioid analgesic misuse: causes, treatments, and policy strategies. Subst Abuse Rehabil.

[3] World Health Organization (2016). International Statistical Classification of Diseases and Related Health Problems 10th Revision (ICD-10).

[4] Jones CM (2013). Heroin use and heroin use risk behaviors among nonmedical users of prescription opioid pain relievers—United States, 2002-2004 and 2008-2010. Drug Alcohol Depend.

[5] Substance Abuse and Mental Health Services AdministrationCenter for Behavioral Health Statistics and Quality. Drug Abuse Warning Network 2011: national estimates of drug-related emergency department visits. Rockville: Substance Abuse and Mental Health Services Administration; 2013. https://www.samhsa.gov/data/sites/default/files/DAWN2k11ED/DAWN2k11ED/DAWN2k11ED.htm.

[6] Canadian Centre on Substance Abuse. Canadian drug summary: prescription opioids, 2015.

[7] Degenhardt L, Hall W (2012). Extent of illicit drug use and dependence, and their contribution to the global burden of disease. Lancet.

[8] Lynskey MT, Fergusson DM (1995). Childhood conduct problems, attention deficit behaviors, and adolescent alcohol, tobacco, and illicit drug use. J Abnorm Child Psychol.

[9] MacCoun R, Kilmer B, Reuter P, Ashcroft J, Daniels DJ, Hart SV (2003). Research on drugs-crime linkages: the next generation. Toward a drugs and crime research agenda for the 21st century.

[10] Roy E, Haley N, Leclerc P (2003). Drug injection among street youths in Montreal: predictors of initiation. J Urban Health.

[11] Coughlin PA, Mavor AID (2006). Arterial consequences of recreational drug use. Eur J Vasc Endovasc Surg.

[12] Schneider GS (2016). Virginia declares opioid emergency, makes antidote available to all.

[13] Dhillon S, Howlett K. B.C. declares public health emergency as overdoses surge again. 2016.

[14] Manchikanti L (2006). Prescription drug abuse: what is being done to address this new drug epidemic? Testimony before the Subcommittee on Criminal Justice, Drug Policy and Human Resources. Pain Physician.

[15] National Opioid Use Guideline Group. Canadian Guideline for Safe and Effective Use of Opioids for Chronic Non-Cancer Pain: recommendations for practice.2010.

[16] Fishbain DA, Cole B, Lewis J (2008). What percentage of chronic nonmalignant pain patients exposed to chronic opioid analgesic therapy develop abuse/addiction and/or aberrant drug-related behaviors?. Pain Med.

[17] Turk DC, Swanson KS, Gatchel RJ (2008). Predicting opioid misuse by chronic pain patients: a systematic review and literature synthesis. Clin J Pain.

[18] Moher D, Shamseer L, Clarke M (2015). Preferred reporting items for systematic review and meta-analysis protocols (PRISMA-P) 2015 statement. Systematic Reviews.

[19] American Society of Clinical Oncology. ASCO Releases Principles for Balancing Appropriate Patient Access to Prescription Opioids with Curbing Misuse, Abuse of these Drugs 2016. Available from: https://www.asco.org/advocacy-policy/asco-in-action/asco-releases-principles-balancing-appropriate-patient-access. Accessed 13 Sept 2017.

[20] Campbell G, Bruno R, Lintzeris N (2016). Defining problematic pharmaceutical opioid use among people prescribed opioids for chronic noncancer pain: do different measures identify the same patients?. Pain.

[21] World Health Organization (2009). Guidelines for the psychosocially assisted pharmacological treatment of opioid dependence.

[22] Shalmi CL. Opioids for nonmalignant pain: issues and controversy. In: Warfield CA, Bajwa ZH, eds. Principles and practice of pain medicine Columbus, OH: McGraw-Hill Companies Inc. 2004:607.

[23] Barnett ML, Olensk AR, Jena AB (2017). Opioid-prescribing patterns of emergency physicians and risk of long-term use. N Engl J Med.

[24] Stroup D, Berlin JA, Morton SC (2000). Meta-analysis of observational studies in epidemiology: a proposal for reporting. Meta-analysis of Observational Studies in Epidemiology (MOOSE) group. JAMA.

[25] Bruehl S, Apkarian AV, Ballantyne JC (2013). Personalized medicine and opioid analgesic prescribing for chronic pain: opportunities and challenges. J Pain.

[26] National Institute for Health and Care Excellence. Methods for the Development of NICE Public Health Guidance (Third Edition). London, United Kingdom. 2012. https://www.ncbi.nlm.nih.gov/pubmedhealth/PMH0089896/pdf/PubMedHealth_PMH0089896.pdf. 27905711

[27] Swarm R, Pickar A, Anghelescu DL (2013). Adult cancer pain. J Natl Compr Cancer Netw.

[28] Ballantyne JC (2007). Opioid analgesia: perspectives on right use and utility. Pain Physician.

