# Supplementary material for: Risk factors for addiction among patients receiving prescribed opioids: a systematic review protocol
Source: Syst Rev. 2017 Dec 28;6:265. doi: 10.1186/s13643-017-0642-0 (PMC5746013; doi:10.1186/s13643-017-0642-0)
Supplement: Additional file 1: — Appendix A. PRISMA-P 2015 Checklist [18]. Appendix B. MEDLINE Search from June 26, 2017. Appendix C. Inclusion/Exclusion Form. Appendix D. Data Collection Form. Appendix E. NICE Quality Appraisal Checklist for Quantitative Studies Reporting Correlations and Associations [26]. Appendix F. NICE Quality Appraisal Checklist for Quantitative Intervention Studies [26]. (DOCX 98.8 kb) [file 13643_2017_642_MOESM1_ESM.docx]

## **Appendix A.** PRISMA-P 2015 Checklist^18^

| Section and topic | Item No | Checklist item | Page number(s) |
| --- | --- | --- | --- |
| ADMINISTRATIVE INFORMATION | | |  |
| Title: |  |  |  |
| Identification | 1a | Identify the report as a protocol of a systematic review | **1** |
| Update | 1b | If the protocol is for an update of a previous systematic review, identify as such | **NA** |
| Registration | 2 | If registered, provide the name of the registry (such as PROSPERO) and registration number | **NA** |
| Authors: |  |  |  |
| Contact | 3a | Provide name, institutional affiliation, e-mail address of all protocol authors; provide physical mailing address of corresponding author | **1** |
| Contributions | 3b | Describe contributions of protocol authors and identify the guarantor of the review | **15** |
| Amendments | 4 | If the protocol represents an amendment of a previously completed or published protocol, identify as such and list changes; otherwise, state plan for documenting important protocol amendments | **NA** |
| Support: |  |  |  |
| Sources | 5a | Indicate sources of financial or other support for the review | **15** |
| Sponsor | 5b | Provide name for the review funder and/or sponsor | **15** |
| Role of sponsor or funder | 5c | Describe roles of funder(s), sponsor(s), and/or institution(s), if any, in developing the protocol | **15** |
| INTRODUCTION | | |  |
| Rationale | 6 | Describe the rationale for the review in the context of what is already known | **4,5** |
| Objectives | 7 | Provide an explicit statement of the question(s) the review will address with reference to participants, interventions, comparators, and outcomes (PICO) | **6,7,8** |
| METHODS | | |  |
| Eligibility criteria | 8 | Specify the study characteristics (such as PICO, study design, setting, time frame) and report characteristics (such as years considered, language, publication status) to be used as criteria for eligibility for the review | **6,7,8** |
| Information sources | 9 | Describe all intended information sources (such as electronic databases, contact with study authors, trial registers or other grey literature sources) with planned dates of coverage | **9,10** |
| Search strategy | 10 | Present draft of search strategy to be used for at least one electronic database, including planned limits, such that it could be repeated | **20,21,22** |
| Study records: |  |  |  |
| Data management | 11a | Describe the mechanism(s) that will be used to manage records and data throughout the review | **10** |
| Selection process | 11b | State the process that will be used for selecting studies (such as two independent reviewers) through each phase of the review (that is, screening, eligibility and inclusion in meta-analysis) | **10,11,23,24** |
| Data collection process | 11c | Describe planned method of extracting data from reports (such as piloting forms, done independently, in duplicate), any processes for obtaining and confirming data from investigators | **10,11,25-28** |
| Data items | 12 | List and define all variables for which data will be sought (such as PICO items, funding sources), any pre-planned data assumptions and simplifications | **6,7,8,25-28** |
| Outcomes and prioritization | 13 | List and define all outcomes for which data will be sought, including prioritization of main and additional outcomes, with rationale | **7,8,25-28** |
| Risk of bias in individual studies | 14 | Describe anticipated methods for assessing risk of bias of individual studies, including whether this will be done at the outcome or study level, or both; state how this information will be used in data synthesis | **12,29-35** |
| Data synthesis | 15a | Describe criteria under which study data will be quantitatively synthesised | **12** |
|  | 15b | If data are appropriate for quantitative synthesis, describe planned summary measures, methods of handling data and methods of combining data from studies, including any planned exploration of consistency (such as I^2^, Kendall’s τ) | **12** |
|  | 15c | Describe any proposed additional analyses (such as sensitivity or subgroup analyses, meta-regression) | **12-13** |
|  | 15d | If quantitative synthesis is not appropriate, describe the type of summary planned | **12** |
| Meta-bias(es) | 16 | Specify any planned assessment of meta-bias(es) (such as publication bias across studies, selective reporting within studies) | **13** |
| Confidence in cumulative evidence | 17 | Describe how the strength of the body of evidence will be assessed (such as GRADE) | **13** |

**APPENDICES**

**Appendix B**. MEDLINE Search from June 26, 2017

Database: Ovid MEDLINE(R) Epub Ahead of Print, In-Process & Other Non-Indexed Citations, Ovid MEDLINE(R) Daily and Ovid MEDLINE(R) <1946 to Present>

Search Strategy:

----------------------------------------------------------------

1 exp Analgesics, Opioid/ad, ae, ct, tu [Administration & Dosage, Adverse Effects, Contraindications, Therapeutic Use] (56486)

2 Analgesics/ (44667)

3 limit 2 to yr="1966 - 1974" (4077)

4 Opioid-Related Disorders/ (10815)

5 Substance-Related Disorders/ (88913)

6 Heroin dependence/ or morphine dependence/ (11921)

7 or/5-6 (98795)

8 limit 7 to yr="1966 - 1979" (14396)

9 (Opioid? adj3 (dependence or misuse or tolerance or disorder or naive)).mp. (5192)

10 Prescription Drug Misuse/ (1090)

11 or/1,3-4,8-10 (80299)

12 pain/ or acute pain/ or breakthrough pain/ or chronic pain/ or earache/ or eye pain/ or flank pain/ or glossalgia/ or labor pain/ or mastodynia/ or neck pain/ or pain, intractable/ or pain, referred/ or renal colic/ (149665)

13 abdominal pain/ or abdomen, acute/ (26925)

14 arthralgia/ or shoulder pain/ (10792)

15 back pain/ or failed back surgery syndrome/ or low back pain/ 34380)

16 chest pain/ or angina pectoris/ or angina, unstable/ or angina pectoris, variant/ or angina, stable/ (53153)

17 pain/ or facial pain/ or toothache/ (133613)

18 headache/ or slit ventricle syndrome/ (26262)

19 metatarsalgia/ or morton neuroma/ (244)

20 musculoskeletal pain/ or myalgia/ or pelvic girdle pain/ (3103)

21 neuralgia/ or morton neuroma/ or neuralgia, postherpetic/ or piriformis muscle syndrome/ or pudendal neuralgia/ or sciatica/ (16977)

22 nociceptive pain/ or visceral pain/ (945)

23 pain, postoperative/ or phantom limb/ (34861)

24 Pelvic Pain/ (4577)

25 or/12-24 (347830)

26 dt.fs. [Drug therapy] (2009552)

27 ep.fs. ^26^ (1451975)

28 or/26-27 (3337592)

29 25 and 28 (107281)

30 Pain Management/ (26682)

31 Pain Clinics/ (1336)

32 ((noncancer or non cancer) adj pain).mp. (1327)

33 ((chronic or acute or severe or extreme) adj3 pain).mp. (88381)

34 or/29-33 (194911)

35 11 and 34 (21760)

36 risk assessment/ (221164)

37 risk factors/ (706690)

38 (risk or risks).mp. (2176328)

39 addiction potential.mp. (172)

40 predict$.mp. (1355393)

41 or/36-40 (3206453)

42 11 and 34 and 41 (3111)

43 animals/ not (human/ and animals/) (4388594)

44 42 not 43 (3060)

45 comment/ or editorial/ or news/ (1173665)

46 44 not 45 (2938)

47 limit 46 to yr="1964 -Current" (2935)

48 limit 47 to (English or French or German) (2874)

49 47 not 48 (61) [Non-English]

## **Appendix C.** Inclusion/Exclusion Form

|  | **Yes** | **No** | | **Comments** |
| --- | --- | --- | --- | --- |
| **Population** |  |  | |  |
| 1. Did the study population consist 100% of patients first exposed to illicit opioids? | **EXCLUDE** | If mixed population, proceed to 1a. | |  |
| - 1. Were patients with illicit versus prescribed opioid initiation analyzed in separate groups? |  | Contact authors & proceed to 1b. | |  |
| - 1. If unable to contact authors for patient-level data, or patient-level data not available, did >50% patients report illicit initiation? | **EXCLUDE** |  | |  |
| 1. Did the study population consist 100% of patients with cancer-related pain? | **EXCLUDE** | If mixed population, proceed to 2a. | |  |
| - 1. Were patients with cancer-related pain analyzed in separate groups from patients with non-cancer-related? |  | Contact authors & proceed to 2b. | |  |
| - 1. If unable to contact authors for patient-level data, or patient-level data not available, did >50% patients report cancer related pain? | **EXCLUDE** |  | |  |
| 1. Did the study population consist 100% of palliative patients? | **EXCLUDE** | If mixed population, proceed to 3a. | |  |
| - 1. Were palliative patients analyzed in separate groups from non-palliative patients? |  | Contact authors & proceed to 3b. | |  |
| 1. If unable to contact authors for patient-level data, or patient-level data not available, were >50% patients palliative? | **EXCLUDE** |  | |  |
| **Outcome**  Uses at least one of the following to define a study outcome: |  | **EXCLUDE** | |  |
| - Pronounced craving for the drug |  |  | |  |
| - Obsessive thinking about the drug |  |  | |  |
| - Erosion of inhibitory control efforts to refrain from drug use |  |  | |  |
| - Compulsive drug taking |  |  | |  |
| - Persisting in use despite harmful consequences |  |  | |  |
| - Higher priority given to drug use than to other activities and obligations |  |  | |  |
| - Increased tolerance |  |  | |  |
| - A physical withdrawal state |  |  | |  |
| - Unintended long-term use |  |  | |  |
| - Higher than intended dose |  |  | |  |
| - Unable to quit |  |  | |  |
| - Spending large quantities of time accessing, using or recovering from use |  |  | |  |
| - Positive urine drug screening |  |  | |  |
| - Any aberrant drug risk behavior: - Selling prescription drugs - Prescription forgery - Stealing or “borrowing” drugs from others - Injecting oral formulations - Obtaining prescription drugs from non-medical sources - Concurrent abuse of alcohol or illicit drugs - Repeated dose escalation or similar noncompliance despite multiple warnings - Repeated visits to other clinicians or emergency rooms without informing prescriber - Drug-related deterioration in function at work, in the family, or socially - Repeated resistance to changes in therapy despite evidence of adverse drug effects - Aggressive complaining about need for more drug - Drug hoarding during periods of reduced symptoms - Requesting specific drugs - Openly acquiring similar drugs from other medical sources - Occasional unsanctioned dose escalation or non-compliance - Unapproved use of drug to treat other symptoms - Reporting psychic effects not intended by clinician - Resistance to change in therapy associated with “tolerable” adverse effects with expressions of anxiety related to the return of severe symptoms |  |  | |  |
| - Diagnosis based on clinical opinion |  |  | |  |
| - Registration to a rehabilitation program |  |  | |  |
| **Topic**  Does the study present data on > 1 risk factor? |  | **EXCLUDE** | |  |
| **Study Design**  Is the study design anything other than a(n) RCT, cross-sectional, case-control, or cohort study? | **EXCLUDE** |  | |  |
|  |  |  |  | |
| **Study Rating** | **EXCLUDE INCLUDE** | | | |

## **Appendix D.** Data Collection Form

| **Study ID:**  *(AuthorSurnameYear)* |  | | | | | |
| --- | --- | --- | --- | --- | --- | --- |
| **Reviewer Initials** |  | | | | | |
| **STUDY METHODS** | | | | | | |
| **Study Design:** | - Cross-sectional - Case-control - Cohort study - Randomized control trial | | | | | |
|  | - Retrospective - Prospective | | | | | |
| **Baseline Sample Size (N)** |  | | | | | |
| **Follow-up Time(s):**  *(length of time between initial opioid exposure and addiction evaluation)* |  | | Sample Size (N) | | Follow-Up Time (days) | |
|  | Follow-Up 1 | |  | |  | |
|  | Follow-Up 2 | |  | |  | |
|  | Follow-Up 3 | |  | |  | |
| **Opioid Addiction Feature(s) Included in Outcome Definition** (*check multiple boxes if applicable*) | - Pronounced craving for the drug - Obsessive thinking about the drug - Erosion of inhibitory control efforts to refrain from drug use - Compulsive drug taking - Persisting in use to despite harmful consequences - Higher priority given to drug use than to other activities and obligations - Increased tolerance - A physical withdrawal state - Unintended long-term opioid use - Higher than intended dose | | | | | |
| **Opioid Addiction Diagnostic Method(s)** (*check multiple boxes if applicable*) | - Clinical opinion - Aberrant drug-risk behaviours (Figure 1) - Urine toxicology - Registration in a rehabilitation program - Administrative data - Chart review - Self-reported - Other (please specify): _________________________________ | | | | | |
| **POPULATION (BASELINE)** |  | | | | | |
| **Country/Countries** |  | | | | | |
| **Recruitment Setting** (*check multiple boxes if applicable*) | - Clinic(s) - Doctor’s Office(s) - Hospital Ward(s) - Emergency Department(s) - Administrative Data - Pain Clinic - Other (please specify): ___________________________________ | | | | | |
| **Mean (or Median) Age at enrolment** *(please specify which and give the measure of variance)* |  | | | | | |
| **Gender** (*male:female*) |  | | | | | |
| **Ethnicity/Race (%)** |  | | | | | |
| **Pain Type** (*check multiple boxes if applicable*) | - Any - Chronic - Acute - Condition-specific (*please specify*): ______________________________ - Other (*please specify*): ______________________________ | | | | | |
| **Baseline Opioid Exposure Status** |  | | Definition used | | Frequency (n) | |
|  | Opioid-naïve (no Rx >1 week) | |  | |  | |
|  | Recently opioid-naïve (no recent Rx >1 week) | |  | |  | |
|  | Opioid-tolerant (Current Rx >1 week) | |  | |  | |
|  | Not sure/Not stated | |  | |  | |
| **Other Baseline Population Descriptors** |  | | Definition used | | Frequency (n) | |
|  | Illicit user (Use with no Rx) | |  | |  | |
|  | Cancer/Palliative | |  | |  | |
|  | Not sure/Not stated | |  | |  | |
| **Other Relevant Population Descriptors** |  | | | | | |
| **RESULTS** | | | | | | |
| **Prevalence of Opioid Addiction Reported** (*include for all possible methods of ascertainment*) |  | | | Frequency (n) | | |
|  | Follow-Up 1 | | |  | | |
|  | Follow-Up 2 | | |  | | |
|  | Follow-Up 3 | | |  | | |
| **Risk Factors (or Risk Factor Sub-category) Investigated**  (+ = ‘Yes’; - = ‘No’) | n (Risk Factor+/ Addiction+) | N (Risk Factor+) | | n (Risk Factor-/Addiction+) | | N (Risk Factor-) |
|  |  |  | |  | |  |
|  |  |  | |  | |  |
|  |  |  | |  | |  |
|  |  |  | |  | |  |
|  |  |  | |  | |  |
|  |  |  | |  | |  |
|  |  |  | |  | |  |
|  |  |  | |  | |  |
|  |  |  | |  | |  |
|  |  |  | |  | |  |
|  |  |  | |  | |  |
|  |  |  | |  | |  |
|  |  |  | |  | |  |
| **ADDITIONAL INFO** | | | | | | |
| **Contact Author for More Information?** | - Yes *🡪 provide contact info and details on what information is missing in Notes below* - No | | | | | |
| **Notes:** | | | | | | |

**Appendix E.**  NICE Quality Appraisal Checklist for Quantitative Studies Reporting Correlations and Associations^26^

| Study identification: Include full citation details |  | |
| --- | --- | --- |
| Study design: |  | |
| Guidance topic: |  | |
| Assessed by: |  | |
| **Section 1: Population** |  | |
| 1.1 Is the source population or source area well described?   - Was the country (e.g. developed or non-developed, type of health care system), setting (primary schools, community centers etc.), location (urban, rural), population demographics etc. adequately described? | ++  +  -  NR  NA | Comments: |
| 1.2 Is the eligible population or area representative of the source population or area?   - Was the recruitment of individuals, clusters or areas well defined (e.g. advertisement, birth register)? - Was the eligible population representative of the source? Were important groups underrepresented? | ++  +  -  NR  NA | Comments: |
| 1.3 Do the selected participants or areas represent the eligible population or area?   - Was the method of selection of participants from the eligible population well described? - What % of selected individuals or clusters agreed to participate? Were there any sources of bias? - Were the inclusion or exclusion criteria explicit and appropriate? | ++  +  -  NR  NA | Comments: |
| **Section 2: Method of selection of exposure (or comparison) group** | | |
| 2.1 Selection of exposure (and comparison) group. How was selection bias minimized?   - How was selection bias minimized? | ++  +  -  NR  NA | Comments: |
| 2.2 Was the selection of explanatory variables based on a sound theoretical basis?   - How sound was the theoretical basis for selecting the explanatory variables? | ++  +  -  NR  NA | Comments: |
| 2.3 Was the contamination acceptably low?   - Did any in the comparison group receive the exposure? - If so, was it sufficient to cause important bias? | ++  +  -  NR  NA | Comments: |
| 2.4 How well were likely confounding factors identified & controlled?   - Were there likely to be other confounding factors not considered or appropriately adjusted for? - Was this sufficient to cause important bias? | ++  +  -  NR  NA | Comments: |
| 2.5 Is the setting applicable to the UK?   - Did the setting differ significantly from the UK? | ++  +  -  NR  NA | Comments: |
| **Section 3: Outcomes** | | |
| 3.1 Were the outcome measures and procedures reliable?   - Were outcome measures subjective or objective (e.g. biochemically validated nicotine levels ++ vs self-reported smoking −)? - How reliable were outcome measures (e.g. inter- or intra-rater reliability scores)? - Was there any indication that measures had been validated (e.g. validated against a gold standard measure or assessed for content validity)? | ++  +  -  NR  NA | Comments: |
| 3.2 Were the outcome measurements complete?   - Were all or most of the study participants who met the defined study outcome definitions likely to have been identified? | ++  +  -  NR  NA | Comments: |
| 3.3 Were all the important outcomes assessed?   - Were all the important benefits and harms assessed? - Was it possible to determine the overall balance of benefits and harms of the intervention versus comparison? | ++  +  -  NR  NA | Comments: |
| 3.4 Was there a similar follow-up time in exposure and comparison groups?   - If groups are followed for different lengths of time, then more events are likely to occur in the group followed-up for longer distorting the comparison. - Analyses can be adjusted to allow for differences in length of follow-up (e.g. using person-years). | ++  +  -  NR  NA | Comments: |
| 3.5 Was follow-up time meaningful?   - Was follow-up long enough to assess long-term benefits and harms? - Was it too long, e.g. participants lost to follow-up? | ++  +  -  NR  NA | Comments: |
| **Section 4: Analyses** | | |
| 4.1 Was the study sufficiently powered to detect an intervention effect (if one exists)?   - A power of 0.8 (i.e. it is likely to see an effect of a given size if one exists, 80% of the time) is the conventionally accepted standard. - Is a power calculation presented? If not, what is the expected effect size? Is the sample size adequate? | ++  +  -  NR  NA | Comments: |
| 4.2 Were multiple explanatory variables considered in the analyses?   - Were there sufficient explanatory variables considered in the analysis? | ++  +  -  NR  NA | Comments: |
| 4.3 Were the analytical methods appropriate?   - Were important differences in follow-up time and likely confounders adjusted for? | ++  +  -  NR  NA | Comments: |
| 4.6 Was the precision of association given or calculable? Is association meaningful?   - Were confidence intervals or p values for effect estimates given or possible to calculate? - Were CIs wide or were they sufficiently precise to aid decision-making? If precision is lacking, is this because the study is under-powered? | ++  +  -  NR  NA | Comments: |
| **Section 5: Summary** | | |
| 5.1 Are the study results internally valid (i.e. unbiased)?   - How well did the study minimize sources of bias (i.e. adjusting for potential confounders)? - Were there significant flaws in the study design? | ++  +  -  NR  NA | Comments: |
| 5.2 Are the findings generalizable to the source population (i.e. externally valid)?   - Are there sufficient details given about the study to determine if the findings are generalizable to the source population? - Consider: participants, interventions and comparisons, outcomes, resource and policy implications. | ++  +  -  NR  NA | Comments: |

NR=not reported, NA=not applicable

**Appendix F.** NICE Quality Appraisal Checklist for Quantitative Intervention Studies ^26^

| Study identification: Include full citation details |  | |
| --- | --- | --- |
| Study design: |  | |
| Guidance topic: |  | |
| Assessed by: |  | |
| **Section 1: Population** |  | |
| 1.1 Is the source population or source area well described?   - Was the country (e.g. developed or non-developed, type of health care system), setting (primary schools, community centres etc), location (urban, rural), population demographics etc adequately described? | ++  +  -  NR  NA | Comments: |
| 1.2 Is the eligible population or area representative of the source population or area?   - Was the recruitment of individuals, clusters or areas well defined (e.g. advertisement, birth register)? - Was the eligible population representative of the source? Were important groups underrepresented? | ++  +  -  NR  NA | Comments: |
| 1.3 Do the selected participants or areas represent the eligible population or area?   - Was the method of selection of participants from the eligible population well described? - What % of selected individuals or clusters agreed to participate? Were there any sources of bias? - Were the inclusion or exclusion criteria explicit and appropriate? | ++  +  -  NR  NA | Comments: |
| **Section 2: Method of allocation to intervention (or comparison)** | | |
| 2.1 Allocation to intervention (or comparison). How was selection bias minimized?   - Was allocation to exposure and comparison randomized? Was it truly random ++ or pseudo-randomized + (e.g. consecutive admissions)? - If not randomized, was significant confounding likely (−) or not (+)? If a crossover, was order of intervention randomized? | ++  +  -  NR  NA | Comments: |
| 2.2 Were interventions (and comparisons) well described and appropriate?   - Were interventions and comparisons described in sufficient detail (i.e. enough for study to be replicated)? - Were comparisons appropriate (e.g. usual practice rather than no intervention)? | ++  +  -  NR  NA | Comments: |
| 2.3 Was the allocation concealed?   - Could the person(s) determining allocation of participants or clusters to intervention or comparison groups have influenced the allocation? - Adequate allocation concealment (++) would include centralized allocation or computerized allocation systems. | ++  +  -  NR  NA | Comments: |
| 2.4 Were participants or investigators blind to exposure and comparison?   - Were participants and investigators – those delivering or assessing the intervention kept blind to intervention allocation? (Triple or double blinding score ++) - If lack of blinding is likely to cause important bias, score −. | ++  +  -  NR  NA | Comments: |
| 2.5 Was the exposure to the intervention and comparison adequate?   - Is reduced exposure to intervention or control related to the intervention (e.g. adverse effects leading to reduced compliance) or fidelity of implementation (e.g. reduced adherence to protocol)? - Was lack of exposure sufficient to cause important bias? | ++  +  -  NR  NA | Comments: |
| 2.6 Was contamination acceptably low?   - Did any in the comparison group receive the intervention or vice versa? If so, was it sufficient to cause important bias? - If a crossover trial, was there a sufficient wash-out period between interventions? | ++  +  -  NR  NA | Comments: |
| 2.7 Were other interventions similar in both groups?   - Did either group receive additional interventions or have services provided in a different manner? - Were the groups treated equally by researchers or other professionals? Was this sufficient to cause important bias? | ++  +  -  NR  NA | Comments: |
| 2.8 Were all participants accounted for at study conclusion?   - Were those lost-to-follow-up (i.e. dropped or lost pre-, during or post- intervention) acceptably low (i.e. typically <20%)? - Did the proportion dropped differ by group? For example, were drop-outs related to the adverse effects of the intervention? | ++  +  -  NR  NA | Comments: |
| 2.9 Did the setting reflect usual UK practice?   - Did the setting in which the intervention or comparison was delivered differ significantly from usual practice in the UK? For example, did participants receive intervention (or comparison) condition in a hospital rather than a community-based setting? | ++  +  -  NR  NA | Comments: |
| 2.10 Did the intervention or control comparison reflect usual UK practice?   - Did the intervention or comparison differ significantly from usual practice in the UK? For example, did participants receive intervention (or comparison) delivered by specialists rather than GPs? Were participants monitored more closely? | ++  +  -  NR  NA | Comments: |
| **Section 3: Outcomes** | | |
| 3.1 Were the outcome measures and procedures reliable?   - Were outcome measures subjective or objective (e.g. biochemically validated nicotine levels ++ vs self-reported smoking −)? - How reliable were outcome measures (e.g. inter- or intra-rater reliability scores)? - Was there any indication that measures had been validated (e.g. validated against a gold standard measure or assessed for content validity)? | ++  +  -  NR  NA | Comments: |
| 3.2 Were the outcome measurements complete?   - Were all or most of the study participants who met the defined study outcome definitions likely to have been identified? | ++  +  -  NR  NA | Comments: |
| 3.3 Were all the important outcomes assessed?   - Were all the important benefits and harms assessed? - Was it possible to determine the overall balance of benefits and harms of the intervention versus comparison? | ++  +  -  NR  NA | Comments: |
| 3.4 Were outcomes relevant?   - Where surrogate outcome measures were used, did they measure what they set out to measure? (e.g. a study to assess impact on physical activity assesses gym membership – a potentially objective outcome measure – but is it a reliable predictor of physical activity?) | ++  +  -  NR  NA | Comments: |
| 3.5 Was there a similar follow-up time in exposure and comparison groups?   - If groups are followed for different lengths of time, then more events are likely to occur in the group followed-up for longer distorting the comparison. - Analyses can be adjusted to allow for differences in length of follow-up (e.g. using person-years). | ++  +  -  NR  NA | Comments: |
| 3.6 Was follow-up time meaningful?   - Was follow-up long enough to assess long-term benefits and harms? - Was it too long, e.g. participants lost to follow-up? | ++  +  -  NR  NA | Comments: |
| **Section 4: Analyses** | | |
| 4.1 Was the study sufficiently powered to detect an intervention effect (if one exists)?   - A power of 0.8 (i.e. it is likely to see an effect of a given size if one exists, 80% of the time) is the conventionally accepted standard. - Is a power calculation presented? If not, what is the expected effect size? Is the sample size adequate? | ++  +  -  NR  NA | Comments: |
| 4.2 Was intention to treat (ITT) analysis conducted?   - Were all participants (including those that dropped out or did not fully complete the intervention course) analysed in the groups (i.e. intervention or comparison) to which they were originally allocated? | ++  +  -  NR  NA | Comments: |
| 4.3 Was the study sufficiently powered to detect an intervention effect (if one exists)?   - A power of 0.8 (that is, it is likely to see an effect of a given size if one exists, 80% of the time) is the conventionally accepted standard. - Is a power calculation presented? If not, what is the expected effect size? Is the sample size adequate? | ++  +  -  NR  NA | Comments: |
| 4.4 Were the estimates of effect size given or calculable?   - Were effect estimates (e.g. relative risks, absolute risks) given or possible to calculate? | ++  +  -  NR  NA | Comments: |
| 4.5 Were the analytical methods appropriate?   - Were important differences in follow-up time and likely confounders adjusted for? - If a cluster design, were analyses of sample size (and power), and effect size performed on clusters (and not individuals)? - Were subgroup analyses pre-specified? | ++  +  -  NR  NA | Comments: |
| 4.6 Was the precision of association given or calculable? Is association meaningful?   - Were confidence intervals or p values for effect estimates given or possible to calculate? - Were CIs wide or were they sufficiently precise to aid decision-making? If precision is lacking, is this because the study is under-powered? | ++  +  -  NR  NA | Comments: |
| **Section 5: Summary** | | |
| 5.1 Are the study results internally valid (i.e. unbiased)?   - How well did the study minimise sources of bias (i.e. adjusting for potential confounders)? - Were there significant flaws in the study design? | ++  +  -  NR  NA | Comments: |
| 5.2 Are the findings generalisable to the source population (i.e. externally valid)?   - Are there sufficient details given about the study to determine if the findings are generalisable to the source population? - Consider: participants, interventions and comparisons, outcomes, resource and policy implications. | ++  +  -  NR  NA | Comments: |

NR= Not Reported, NA=Not Applicable
